# Spaceflight and simulated microgravity conditions increase virulence of *Serratia marcescens* in the *Drosophila melanogaster* infection model

**DOI:** 10.1038/s41526-019-0091-2

**Published:** 2020-02-04

**Authors:** Rachel Gilbert, Medaya Torres, Rachel Clemens, Shannon Hateley, Ravikumar Hosamani, William Wade, Sharmila Bhattacharya

**Affiliations:** 1 NASA Postdoctoral Program, Universities Space Research Association, NASA Ames Research Center, Moffett Field, CA USA; 2 FILMSS/Bionetics, NASA Ames Research Center, Moffett Field, CA USA; 3 Department of Molecular and Cell Biology, University of California, Berkeley, CA USA; 4 NASA Ames Research Center, Moffett Field, CA USA

**Keywords:** Infection, Innate immunity, Microbiology

## Abstract

While it has been shown that astronauts suffer immune disorders after spaceflight, the underlying causes are still poorly understood and there are many variables to consider when investigating the immune system in a complex environment. Additionally, there is growing evidence that suggests that not only is the immune system being altered, but the pathogens that infect the host are significantly influenced by spaceflight and ground-based spaceflight conditions. In this study, we demonstrate that *Serratia marcescens* (strain Db11) was significantly more lethal to *Drosophila melanogaster* after growth on the International Space Station than ground-based controls, but the increased virulence phenotype of *S. marcescens* did not persist after the bacterial cultures were passaged on the ground. Increased virulence was also observed in bacteria that were grown in simulated microgravity conditions on the ground using the rotating wall vessel. Increased virulence of the space-flown bacteria was similar in magnitude between wild-type flies and those that were mutants for the well-characterized immune pathways *Imd* and *Toll*, suggesting that changes to the host immune system after infection are likely not a major factor contributing towards increased susceptibility of ground-reared flies infected with space-flown bacteria. Characterization of the bacteria shows that at later timepoints spaceflight bacteria grew at a greater rate than ground controls in vitro, and in the host. These results suggest complex physiological changes occurring in pathogenic bacteria in space environments, and there may be novel mechanisms mediating these physiological effects that need to be characterized.

## Introduction

As space exploration extends beyond low Earth orbit, there is considerable interest in understanding the changes in human physiology under spaceflight conditions. Spaceflight includes not only long-term exposure to microgravity, but also radiation, isolation, and short-term exposure to hypergravity. Previous studies have demonstrated that these stressors have a significant impact on the ability of the immune system to respond appropriately to disease, including altered cytokine production,^1–5^ changes in immune cell proliferation and distribution^4,6,7^ and general alteration of immune homeostasis in both innate and adaptive immune systems.^8,9^

This apparent immune dysregulation is even more important when considering that there have been studies demonstrating significant effects of microgravity and spaceflight conditions on the pathogens that co-occur with humans in space habitats.^10–17^ Opportunistic pathogenic bacteria, such as those that can cause persistent infections in immunocompromised individuals, have been shown to have increased growth in spaceflight,^18,19^ increased antibiotic resistance, and increased virulence.^20,21^

Investigations of the effects of spaceflight in humans have been especially varied, likely due to both low sample sizes and variability in experimental methodology.^22^ The fruit fly (*Drosophila melanogaster*) is a promising model for understanding the effects of spaceflight on human immunity and pathogenesis, as they have been shown previously to experience a dramatic shift in immune gene expression following spaceflight.^23,24^ Using invertebrate models for investigating the human immune system has proven both fruitful and convenient, as they are cost-effective, have short generation times, and importantly, have immune systems with high homology to humans.^25,26^ While invertebrates lack a characterized adaptive immune system, the inducible innate immune response of *Drosophila melanogaster* is highly similar to humans,^25,27,28^ with about 75% of human disease-causing genes with a functional homolog in the fruit fly.^25,29^

The human opportunistic bacteria *Serratia marcescens* has only recently emerged as a model for studying rapid shifts in virulence and antibiotic resistance due to increased nosocomial reports.^30,31^ Furthermore, *S. marcescens* has been found aboard the spacecraft Mir, and in condensed water aboard the International Space Station (ISS),^32,33^ suggesting that this ubiquitous pathogen should be monitored closely for spaceflight-induced changes that may pose a threat to immunocompromised astronauts who could be more susceptible to opportunistic pathogens. *D. melanogaster* has been recently used as a model for studying *S. marcescens*, as there are bacterial strains that have been isolated from wild-caught flies that are pathogenic.^34–38^ Importantly, the specific strains used in this study are not pathogenic to humans,^34^ making this system an ideal model for safely studying spaceflight-induced changes to *S. marcescens* using a model system.

Studies in true spaceflight conditions are difficult to perform due to cost, limited availability of launches and crew time, and the complexity of experimental design under the tight volumetric and mass constraints of space habitats. Consequently, many studies on the effects of spaceflight on bacteria are also followed up with ground-based simulated microgravity experiments,^16,17,39^ which allows for further investigation into microgravity-related effects. In this study, the opportunistic pathogen *Serratia marcescens* was sent to the ISS, where it was exposed to true spaceflight conditions, while ground-based strains were used in tandem both as controls and as replicates grown in simulated microgravity conditions (rotating wall vessel (RWV) apparatus) in order to investigate whether changes in bacterial physiology could be replicated accurately in future ground-based studies. In our study, space-flown or simulated microgravity-treated bacteria were used to infect *D. melanogaster* to investigate changes in space-induced lethality, both in wild-type flies, as well as in immune pathway mutants. Results showed that both space-flown and ground-based microgravity simulations (e.g., low-shear modeled microgravity or LSMMG treatment) resulted in increased virulence of *Serratia marcescens* on *D. melanogaster* hosts. Interestingly, the increased virulence phenotype is lost after passaging of the space-flown and LSMMG-treated bacteria under ground conditions. Taken together, this suggests that spaceflight induces a physiological change in the pathogen resulting in the decreased survivability of the *D. melanogaster* host and that this effect can be reversed after the bacterial cells resume normal growth on the ground.

## Results

### Survival of *D. melanogaster* after infection with spaceflight samples

**Fig. 1:**
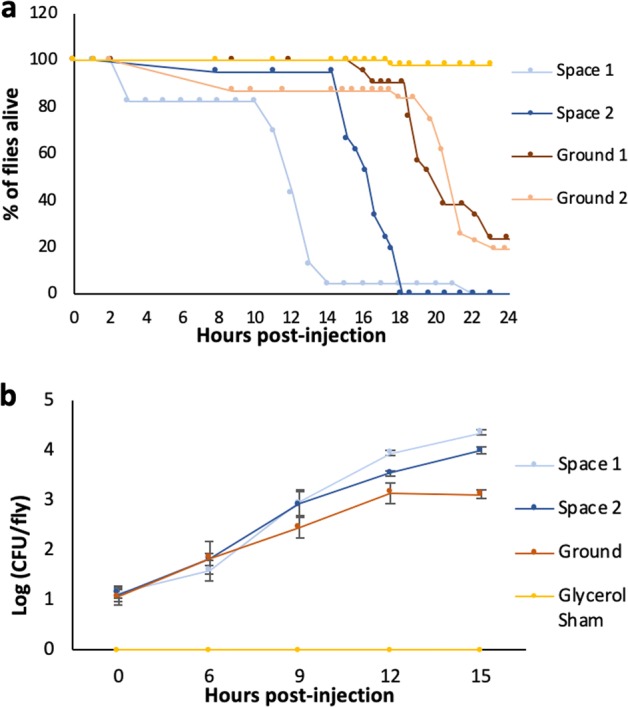
Spaceflight-exposed *Serratia marcescens* show increased virulence in flies. **a** Graph showing survival of *w*^*1118*^ flies after injection with ground or spaceflight Db11 samples. **b** Graph showing in vivo growth of Db11 after injection into *w*^*1118*^ flies. Error bars represent one standard error.

Fly survival was significantly lower when infected with spaceflight-exposed *Serratia marcescens* samples compared to the ground control bacterial samples (Fig. 1). For the first spaceflight sample (Space 1), survival was significantly different from both Ground 1 (*P* < 0.0001) and Ground 2 (*P* < 0.0001) samples. The same was true for the second spaceflight isolate (Space 2) when compared with ground control samples. Full statistical results are reported in Table 1. Space 1 and Space 2 were separate samples of bacteria collected from two different plates after spaceflight, and both showed increased virulence compared to ground control bacteria. However they also showed differences between each other in terms of the magnitude of their changes with Space 1 causing greater virulence changes compared to Space 2 samples. This is discussed later in the context of temporary/reversible physiological changes induced by spaceflight vs genetic/heritable changes in the bacteria (see below for “Host survival after injection of first ground subculture of spaceflight sample”).

**Table 1:** Statistical analysis for Fig. 1a.

Level 1	Level 2	Ratio	*P* > Chi-square
Space 1	12.5% Glycerol	3.15	<0.0001
Space 1	Ground 1	6.64	<0.0001
Space 1	Ground 2	5.69	<0.0001
Space 1	Space 2	1.15	0.68
Space 2	12.5% Glycerol	2.74	<0.0001
Space 2	Ground 1	5.77	<0.0001
Space 2	Ground 2	4.95	<0.0001
Ground 1	12.5% Glycerol	4.73	<0.0001
Ground 2	12.5% Glycerol	5.53	<0.0001
Ground 2	Ground 1	1.16	0.628

Statistical results of the Cox Proportional Hazards test for the spaceflight sample survival analysis shown in Fig. 1a. ‘Ratio’ refers to the ratio of the hazard rates corresponding to the conditions described by Level 1 and Level 2, respectively. This value can be interpreted as a magnitude of differences in survival between Level 1 and Level 2

### In vivo growth of spaceflight *S. marcescens* in fly host after injection

In vivo growth of injected bacteria was not significantly different between space and ground samples at hours 0 (*F* = 0.9113, *P* = 0.489), 6 (*F* = 0.5316, *P* = 0.6772) or 9 (*F* = 1.169, *P* = 0.3964) post-injection. At hour 12 post-injection, in vivo growth of Space 1 sample was significantly higher in the fly host than the Ground sample (*P* = 0.0002) and Space 2 (*P* = 0.0011). At hour 15 post-injection, in vivo growth of Space 1 remained significantly higher than Ground (*P* = 0.0002) and Space 2 (*P* = 0.04), and Space 2 in vivo growth was significantly higher than Ground (*P* = 0.006) (Fig. 1b). Therefore, the kinetics of bacterial growth within the host are different for the bacterial cultures returning from space compared to the ground control samples, particularly 12 h and onwards post-infection. In vitro growth of the space-flown bacteria, outside the host, show a similar increase in growth kinetics at later time points compared to the ground control bacteria (Supplementary Fig. 1).

### Host survival after injection of first ground subculture of spaceflight sample

**Fig. 2:**
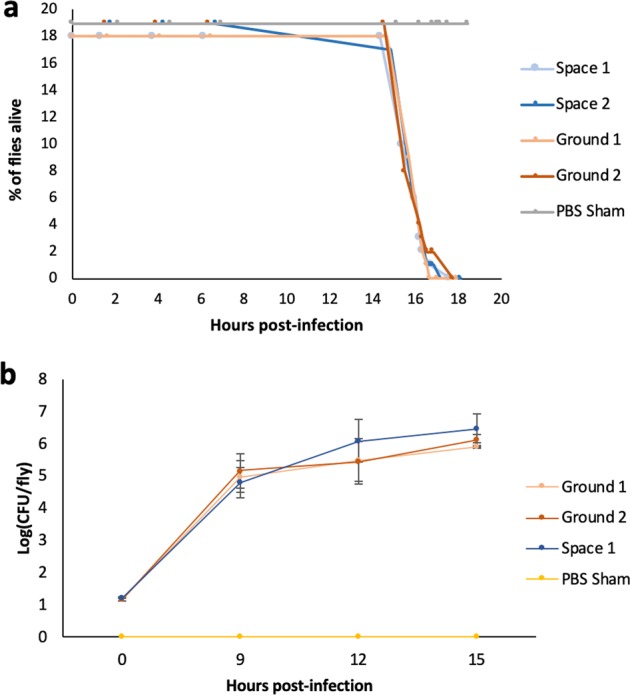
Increased virulence of spaceflight-exposed *S. marcescens* is reversible on the ground. **a** Graph showing survival of *w*^*1118*^ flies after injection with first subcultures of ground or spaceflight Db11 samples. **b** Graph showing in vivo growth of Db11 after injection into *w*^*1118*^ flies. There was no significant difference in survival of *w*^*1118*^ flies after injection with first subcultures, nor was there significant difference revealed in the assay for in vivo bacterial growth. Error bars represent one standard error.

Given that two separate isolates of bacteria had shown increased virulence after spaceflight exposure (Fig. 1), we wanted to assess whether these changes were reversible physiological changes in the bacteria induced by the stress of spaceflight or whether they were genetically heritable changes induced, for instance, by ionizing radiation exposure during spaceflight. After subculturing spaceflight and ground samples of *S. marcescens* in normal Earth gravity overnight, Space 1-injected host survival was not significantly different from either Ground 1 (*P* = 0.995) or Ground 2 (*P* = 0.337) host injected samples. Similarly, Space 2 subculture-infected hosts did not show significant differences in survival from Ground 1 (*P* = 0.5223) or Ground 2 (*P* = 0.113) infected hosts (Fig. 2a). Full statistical output is reported in Table 2. These results suggest that the increased virulence detected after spaceflight were temporary changes acquired by the bacteria during spaceflight and that after subculturing under normal conditions on Earth, these differences were no longer detectable after infection of fruit fly hosts.

**Table 2:** Statistical analysis for Fig. 2a.

Level 1	Level 2	Ratio	*P* > Chi square
Space 1	PBS Sham	21.517	<0.0001
Space 2	PBS Sham	15.661	<0.0001
Ground 1	PBS Sham	21.565	<0.0001
Ground 2	PBS Sham	17.671	<0.0001
Space 1	Ground 1	0.997	0.995
Space 1	Ground 2	1.803	0.337
Space 2	Ground 1	0.7262	0.5223
Space 2	Ground 2	2.0402	0.1131

Statistical results of the Cox Proportional Hazards test for the first ground subculture of spaceflight sample survival analysis shown in Fig. 2a. ‘Ratio’ refers to the ratio of the hazard rates corresponding to the conditions described by Level 1 and Level 2, respectively. This value can be interpreted as a magnitude of differences in survival between Level 1 and Level 2

### In vivo growth after injection of first ground subculture of spaceflight sample

There was no significant difference of in vivo growth of the spaceflight vs ground bacteria within the host (Fig. 2b) at hours 0 (F = 13.41, *P* = 0.146), 9 (*F* = 0.238, *P* = 0.866), 12 (*F* = 0.443, *P* = 0.731) or 15 (*F* = 0.479, *P* = 0.708) hours post-infection. This data was consistent with the fact that there were no differences detected in virulence of the first ground subcultures in the host (Fig. 2a).

### Host survival after injection with LSMMG-treated bacteria

**Fig. 3:**
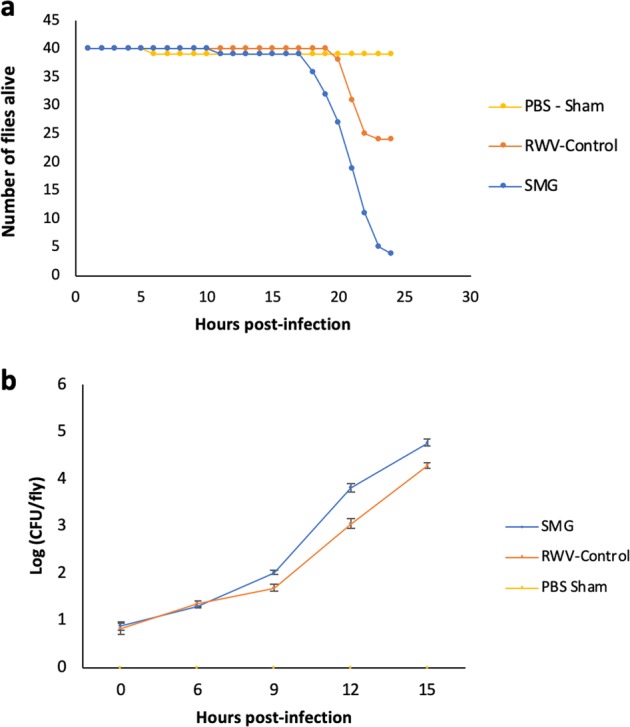
Simulated microgravity (SMG) can increase virulence of *S. marcescens*. **a** Graph showing survival of *w*^*1118*^ flies after injection with LSMMG-treated (SMG), RWV control-treated or sham-treated Db11 samples, and **b** Graph showing in vivo growth of LSMMG-treated (SMG), RWV control or sham-treated Db11 after injection into *w*^*1118*^ flies. Error bars represent one standard error.

Since spaceflight opportunities are relatively infrequent, a model that has often been used on the ground to simulate microgravity conditions for aqueous cultures is the high aspect ratio vessel (HARV) or the RWV.^15,17,39^ We used the RWV apparatus to assess whether we could recreate some or all of the virulence changes that we had measured from the spaceflight samples by simulating the low fluid shear of the microgravity environment on Earth. This is done by rotating the vessel in a perpendicular orientation, where the rotation of the apparatus offsets the sedimental effects of the gravity force and provides continual suspension of the cells. In the control, the axis of rotation is parallel with the gravity force vector and as such, this force is not offset and the cells are allowed to settle.^11,13^ Host survival was significantly lower for hosts infected with *S. marcescens* that was grown in LSMMG compared to both the PBS Sham (ratio = 4.42, *P* < 0.0001) and the RWV Control (Ratio = 3.37, *P* = 0.0002) (Fig. 3a). The RWV Control consists of the same container as is used to simulate microgravity but is rotated about an axis that is oriented at 90 degrees to the LSMMG rotation and is therefore exposed to 1 g Earth’s gravity.^11^ Figure 3a therefore shows that we are able to recreate increased virulence in *S. marcescens* bacteria by simulating microgravity on Earth. This would suggest that the microgravity aspect of the spaceflight environment is at least one important factor to consider in terms of increased virulence in *S. marcescens*.

### In vivo growth of LSMMG *S. marcescens*

There was no significant difference of in vivo growth between the LSMMG and RWV-Control bacteria at hour 0 (*F* = 3.551, *P* = 0.128) or at hour 6 post-infection (*F* = 7.493, *P* = 0.0544). However, there was a significant difference in CFU counts per fly between the LSMMG and RWV-Control at hours 9 (*F* = 20.428, *P* = 0.008), 12 (*F* = 12.455, *P* = 0.019), and 15 (*F* = 18.427, *P* = 0.009) post-infection (Fig. 3b). These altered growth kinetics also mirror some of the differences observed previously with the spaceflight samples (Fig. 1b), where the bacterial samples that show increased virulence, also show increased rates of growth within the host particularly at later time points.

### Infection of Immune Deficiency (*Imd*) and *Toll* pathway mutants of *D. melanogaster*

**Fig. 4:**
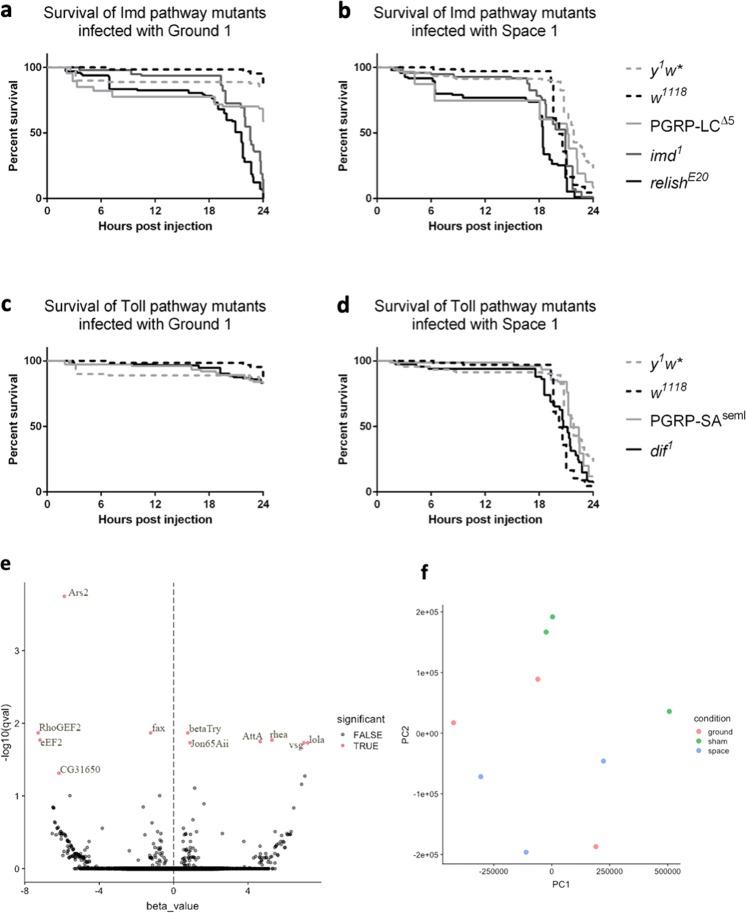
Measuring host changes in wild-type and mutant ground flies exposed to space-flown *S. marcescens*. Survival curves of *Imd* pathway mutant flies, **a** and **b**, and of *Toll* pathway mutant flies, **c** and **d**, after exposure to either Ground 1 bacteria, **a** and **c**, or Space 1 bacteria, **b** and **d**. All graphs have data from two wild-type flies (*y*^*1*^w^*^ and *w*^*1118*^) for reference. **e** Volcano plot of the RNA-seq data from ground-reared wild-type *D. melanogaster* that were infected with space bacteria relative to ground bacteria. RNA was extracted from infected flies 18 h post-infection. The *x*-axis represents the beta-value, an estimator of fold change, and the y-axis represents the −log10*q* value, a *p*-value adjusted for false discovery rate. Red dots represent transcripts with *q*-value < 0.05, which are considered significantly differentially expressed. **f** PCA of ground, space, and sham injected flies shows no major difference in distribution of the groups between the conditions, with as much variation occurring within conditions as between conditions.

Since the increase in virulence of spaceflight bacteria was accompanied by an increase in the rate of growth of the bacteria within the fly host, we wanted to determine whether this was a result of a change that the bacteria were able to induce in the host by weakening the hosts’ immune system for example, or whether the bacterial physiology was altered to better withstand the host’s immune defenses. In order to test this hypothesis, we used flies that were mutant in two of the major immune defense pathways in flies, the *Imd* and the *Toll* pathways.^40,41^ As expected, all *Imd* mutant flies (*PGRP-LC*^*Δ5*^*, imd*^*1*^, and *relish*^*E20*^) succumb to any *Sm* infections (including ground control *Sm*) much more quickly than either of the wild-type fly lines (*y*^*1*^w* or *w*^*1118*^*)* as seen in Fig. 4a, since the *Imd* pathway is known to be critical for defense against infection with Gram-negative bacteria,^42^ such as *Serratia marcescens*. On the other hand, the *Toll* pathway is primarily known to be important for defense against Gram-positive bacterial and fungal infections. Therefore as expected, there is much less change in the viability of *Toll* deficient mutants (*PGRP-SA*^*seml*^ and *dif*^*1*^) after ground control *Sm* infections compared to wild-type flies (Fig. 4c). Compared to ground control *Sm* however, the spaceflight bacteria Space 1 causes greater lethality in all of the fly lines (compare Fig. 4b vs a, and 4d vs c). Therefore the data suggests that regardless of the genotype of the fly host, whether impaired for immune function or robust wild-type lines, the space-flown bacteria causes greater lethality than ground control *Sm*. The statistical output is shown in Supplementary Tables 2–6.

### RNA sequencing data analysis of infected ground-reared host

To further test whether infection with microgravity-treated bacteria induced changes in gene expression in the ground-reared host, RNA sequencing was performed for wild-type fruit flies after infection with either spaceflight or ground control sample of *S. marcescens*. These results showed that there were few significant changes in host gene expression of known immune-related genes when exposed to the two bacterial sample types (Fig. 4e, Supplementary Table 1).

At the significance threshold (*q*-values < 0.05), only eleven transcripts were significantly differentially expressed. However principal components analysis (PCA) comparing transcript abundance across the treatments showed that there is significant overlap among the groups, further indicating that there are few differences between the space bacteria-infected and ground bacteria-infected fruit fly hosts (Fig. 4f). It is worth noting that, since the flies collected for RNA-Seq were still alive at 18 h post-injection, this may have biased the selection of flies in which infection had not greatly progressed, as they were still alive at this late time point. Similarity in gene expression across both conditions to a control sham condition shown by PCA supports this idea.

Comparing the space bacteria-infected ground flies to the ground bacteria-infected flies, there were a total of 11 differentially expressed genes (see Fig. 4e and Supplementary Table 1 for gene details). Out of these 11 differentially expressed genes, only *AttA* (Attacin-A) is known to be directly related to the immune response.^43^ The gene *AttA* is an antimicrobial peptide that is expressed in the fat body in response to Gram-negative pathogens.^43^ The most highly overexpressed gene is *lola* (longitudinals lacking), a gene that is involved in axon growth and guidance in the central and peripheral nervous systems.^44^ The next most highly overexpressed gene is *vsg* (visgun), a gene linked to alcohol sensitivity.^45^ Among the most underexpressed genes is *eEF2*, is a translation elongation factor,^46^ and CG31650 with unknown function. The underexpressed *RhoGEF2* has been recently linked to wound repair, but is primarily involved in the regulation of actin and myosin dynamics through interacting effector proteins.^47^ While only one of these genes is directly related to the host immune pathway, follow-up studies will be conducted to elucidate the potential role of these additional differentially expressed genes in the host.

## Discussion

In this study, we found that flies infected with a bacterial pathogen that had been grown during spaceflight was significantly more lethal than ground controls (Fig. 1). This trend persisted in strains that had been grown in LSMMG conditions, suggesting that microgravity may be the primary factor mediating the differences in virulence seen in the spaceflight bacteria strains (Fig. 3). Interestingly, this increased lethality disappears after the first subculture is conducted on the ground, which means that the differences in virulence are short-lived and do not persist after being cultured under normal gravity conditions (Fig. 2). This is consistent with previous studies, which found that spaceflight-induced changes to pathogens, such as *Bacillus subtilis; Escherichia coli; Pseudomonas aeruginosa; Staphylococcus aureus* reverted upon return to Earth conditions.^10^

We also sought to understand whether the host immune system may be responding differently to spaceflight-altered bacteria. While it has been shown in previous studies that space-flown pathogens decrease the survival of hosts compared to their ground-reared counterparts,^15,16^ less is known about how the host immune system may be influenced differently by space-flown pathogens. This is also the first study in spaceflight and in simulated microgravity to evaluate microgravity-induced changes of the pathogen *S. marcescens* in a live host, as previous studies in the field of host-pathogen interactions have focused primarily on *Salmonella*^15^ and other microbes.^48–50^ Utilizing common immune pathway mutants, we determined that regardless of whether the *Imd* or *Toll* pathways were altered, infection with spaceflight sample decreases survival of flies compared to infection with ground sample of *S. marcescens* (Fig. 4). Studies in spaceflight and modeled microgravity have shown that when the host is exposed to microgravity conditions, the immune responses can be altered by the presence of symbiotic bacteria.^51^ Other studies have shown that when different pathogens are exposed to LSMMG and then tested on naive hosts, responses can vary widely depending on the specific pathogen used.^52^ Similarly in our study with ground-reared fly host, while there appear to be few genes that are differentially expressed after injection with spaceflight-exposed *Serratia marcescens* compared to the ground control bacteria, most of the highly differentially regulated genes do not appear to be directly immune-related, with the exception of Attacin (Fig. 4c, Supplementary Table 1). This antimicrobial peptide is known to respond to Gram-negative pathogens,^43^ so an increase in expression in response to *S. marcescens* is expected. However, the increase in expression in space-infected flies compared to ground-infected flies may be due to the increase in bacterial load in the space-infected individuals.

Because there is a significant increase of in vivo growth seen in both spaceflight samples and LSMMG samples, it may be possible that some of these host genes may have an unknown interaction with the immune system that facilitates the faster proliferation of microgravity-experienced bacteria after infection. However given that the space-flown bacteria have faster growth kinetics in vitro (Supplementary Fig. 1) as well as in vivo, the current results indicate that the differences in lethality and survival following infection of bacteria altered by spaceflight and LSMMG are likely due to changes to the pathogen, rather than a difference in host immune response of ground-reared flies.

This study is the first to demonstrate that spaceflight influences the growth and virulence characteristics of bacteria grown on a solid substrate. While many previous studies have shown that cell density and growth characteristics are altered in liquid suspension during spaceflight (reviewed in Huang et al.,^53^) the few studies that have cultured microbes on solid media found no difference in final cell density.^54^ Therefore, it was concluded that because there is no change in gravity vector for bacteria that are attached to a surface compared to those in a liquid suspension where there would be a lack of convection, that one should not expect to see a change in phenotype associated with growth on solid media in spaceflight. This argument is based on the idea that the bacteria are being affected indirectly by microgravity, due to the change in the physical properties of the liquid media that they are in, and not the effect of a change in gravity vector on the microbes themselves.^55^

However, *Serratia marcescens* is a nosocomial pathogen that can colonize a wide variety of habitats, and undergo significant phenotypic shifts depending on the properties of the growth media,^56–58^ including shifts in the polarity of the flagella on solid media to produce swarming cells. Because it’s thought that LSMMG may influence cell polarity in some single-celled organisms, it’s at least theoretically possible that some of these species may still be affected by microgravity at the cellular level, even on a solid substrate.^59^ A previous study showed that growth of *E.coli* and *B.subtilis* on agar in spaceflight did not show increased growth rates, and they did not measure virulence phenotypes of the samples.^54^ Therefore, it is possible that changes in phenotype in space can be manifested differently in different bacteria depending on the inherent characteristics required to colonize their normal habitat.

While Wang et al.^60^ showed that *S. marcescens* did not change in physical properties on a semi-solid media in spaceflight conditions, they did provide evidence that significant transcriptomic and proteomic changes related to metabolism occurred in spaceflight sample compared to ground. We believe that these molecular and biological changes observed in *S. marcescens* on a semi-solid media in spaceflight provide a basis for the phenotypic changes observed in our study with bacteria grown on solid semi-defined media. While there was insufficient bacterial sample to conduct transcriptomic and proteomic analyses in the current study, a subsequent spaceflight experiment in our lab has been conducted with *S. marcescens* to further characterize molecular changes on both solid and liquid media types in space and will be reported separately. This indicates that media type should not be limited to liquid suspension, and that further studies using both solid and liquid media types is warranted. A study performed with a different strain of *S. marcescens* (ATCC 14041) in LSMMG in liquid media showed that while there was no significant change in growth parameters, there was an increase in acid stress resistance, providing further evidence for phenotypic changes of this species to microgravity and LSMMG.^61^ Ongoing work in our lab, including more in-depth characterization of *S. marcescens* under LSMMG and spaceflight conditions, are currently being conducted to further elucidate the potential molecular and physiological mechanisms affecting growth kinetics and changes in virulence seen in altered gravity.

A previous study found that growth rate was not a major factor affecting the physiology and virulence of one strain *S. marcescens*,^62^ but there is speculation that growth rate and virulence may be linked due to common factors that influence both characteristics.^63,64^ Recent studies in humans demonstrated that slower growth rates of *Bacillus* pathogens actually predicted higher virulence,^65^ suggesting that there is conflicting data that likely varies by species and environmental factors and that growth rates may be linked to virulence. In the current study, not only was the in vitro growth at later time points significantly higher in the spaceflight strains than in the ground control strains (Supplementary Fig. 1), and there is a significant difference with in vivo growth after infection with spaceflight and ground control strains (Fig. 1b), as well as increased in vivo growth in LSMMG bacteria compared to the RWV-control (Fig. 3b). This suggests that bacteria growth rates are perhaps linked to the increased lethality between spaceflight strains and ground strains, especially since we did not find significant changes in host immune-related genes after infection with spaceflight sample compared to ground sample in wild-type ground-reared flies (Fig. 4a–f and Supplementary Table 1).

Furthermore, in vitro growth was significantly higher for spaceflight samples compared to ground samples, specifically starting around hour 12, which is consistent with the in vivo increase in growth after injections (Supplementary Fig. 1). Previous studies have shown that the media composition and nutrient availability can have significant effects on growth kinetics and virulence of pathogens.^16^ Current studies in our laboratory are testing whether microgravity treatment confers a specific growth advantage over ground bacteria at later times in growth when specific nutrients may become limiting in the media, as well as whether or not growth kinetics can be affected by the inclusion or exclusion of specific growth factors from the media. Specifically, it may be possible that the spaceflight and LSMMG environment may confer some advantage to the bacteria when nutrients become limited, such that the low fluid shear environment allows growth to either increase or not become hindered by the decrease in nutrient in the media over time.

This also emphasizes the need to understand the underlying molecular mechanisms of pathogens that are changing in LSMMG and spaceflight conditions, which has become a field of great interest recently. It has been shown that after spaceflight, there are global changes in expression of genes that may potentially be related to virulence^15,17^ and that some pathogens are more likely to form biofilms after exposure to spaceflight.^18^ Because the pathogens in the current study are also Gram-negative and are similarly opportunistic to the pathogens evaluated in previous studies, it may be expected that there are quantifiable genetic mechanisms that may be responsible for the decreased survival we found after infection. Therefore, current studies are focused on eliciting the potential underlying genetic mechanisms that are driving increased virulence.

## Materials and methods

### *D. melanogaster* fly strains

Fly lines were maintained in a 12-h light-dark cycle on cornmeal-agar media (torula yeast, dextrose, cornmeal, agar, Tegosept, propionic acid, ethanol) at ambient temperature (~24 °C). All fly lines were obtained from Bloomington Stock center including *w*^1118^ (#3605), OregonR SP2 (no. 2376), relE20 (no. 55714), y^1^w* (no. 55707), *dif*^1^ (no. 36559), *imd*^1^ (no. 55711), *PGRP-LC*^Δ5^ (#36323), and *PGRP-SA*^seml^ (#55716).

### Bacterial growth conditions during spaceflight

The hardware used in this study was the FIT (Fly Immunity and Tumors) cassette and changeout platform, as described in our previous *Drosophila* experiments on the ISS.^23,66^ Food trays were prepared using the methods described below, and stored in a cargo transfer bag (CTB) at 4 °C for launch. Towards the end of the 30 day spaceflight (Fruit Fly Lab – 01 on SpaceX-CRS 5 mission), the food trays containing *S. marcescens* were retrieved from 4 °C storage and placed within the FIT food changeout platform. The platform was placed in a canvas CTB at ISS ambient temperature (23–25 °C) for 5 days, plus 2 days in the SpaceX Dragon (22–25 °C) for undocking/descent and retrieval. During the growth period, environmental data (CO_2_, O_2_, temperature, and relative humidity) was recorded and used to match the growth conditions for ground control experiments.

### Preparation of *S. marcescens* Db11 for spaceflight

*Serratia marcescens* Db11 was obtained from the University of Minnesota *C. elegans* stock center. To prepare for the spaceflight experiment, the Db11 strain was grown for 20 h in liquid LB broth with 100 µg/mL streptomycin at 37 °C and then diluted to an OD600 of 0.100 in PBS. Next, 100 µL of the diluent was spread onto the surface of two trays containing 2 mL of solid semi-defined fly food media containing 4% Brewer’s yeast (Redstar), 6% glucose, 3% sucrose, 2% yeast extract, 2% peptone, 1% agar, 0.05% magnesium sulfate heptahydrate, 0.05% calcium chloride dihydrate (Sigma), 0.5% of blue food dye (Smart and Final), 0.001% p-hydroxybenzoate, and 0.6% of propionic acid (Sigma) dissolved in water. These two trays were the source of the Space 1 and Space 2 samples that were used in the experiments below. Similarly, we prepared two additional trays (Ground 1 and Ground 2) from the same source sample. These two trays were grown in the same FIT hardware on the ground, inside an incubator (Percival model DR36VL), which was programmed to match the gas composition, temperature, and humidity profiles measured during the flight.

After preparing the bacterial sample trays for flight, they were placed into plastic bags and stored at 4 °C, then sent to the ISS on SpaceX CRS-5. Control trays were stored on the ground at 4 °C. Growth conditions on the ISS were described in the previous section. Upon retrieval after the spaceflight mission, bacteria were isolated from the surfaces using sterile cell scrapers and then placed into 50% glycerol and stored frozen at −80 °C. Similar isolation was performed in parallel for the ground samples.

The placement of the bacterial cultures on semi-defined fly food media allowed for inflight infection of flies, as flies would ingest the bacteria that was growing on the media. However, due to logistical constraints during flight, in-flight infection was not possible and the bacterial cultures were grown in space and harvested after return for treatment of flies on the ground after flight as described in the following sections.

### Injection of spaceflight *S. marcescens* Db11 samples into *D. melanogaster*

Injections of bacteria were performed according to methods described previously by our group and others.^23,67^ Concentrations of spaceflight and ground samples stored in 50% glycerol at −80 °C were determined by performing colony counts on LB plates. These samples were then diluted in PBS (phosphate buffered saline) down to ~4 × 10^3^ CFU/mL in sterile PBS for injections. Sterile 12.5% glycerol in sterile PBS was used as a sham injection control. For survival experiments and in vivo growth assays for both spaceflight bacteria and ground-based simulated microgravity-treated bacteria, the Nanoject II (Drummond Scientific) was used with the 32 nL injection volume setting (Figs 1–4). Concentrations were adjusted so that each injection was ~10 CFU/fly. Control, ground, or spaceflight samples were then injected into anesthetized 2–3 day-old female (post-eclosion) *D. melanogaster* in the ventrolateral abdomen. Injections of immune mutant flies were done using a Picospritzer III (Parker Hannifin, New Hampshire). Injection bolus was calibrated to 600 nm diameter, which equaled approximately 10 CFU/fly. Injections of each treatment group took <1 h, with the survival measurements beginning at the end of that first hour. After injections, flies were kept at 25 °C on the same standard dextrose food that they were reared on, and survival was monitored every hour for at least 24 h. Survival curves were measured by performing three independent experiments using a minimum of *N* = 50 flies in each experiment, then pooling the data from all three experiments for the Cox Proportional Hazard survival analysis, which measures the magnitude of difference in survival between different treatment groups.

In vivo bacterial load was assayed in the fly at 0, 9, 12, and 15 h post-injection. Bacterial load was assayed immediately following injection by placing three flies in 200 µL of PBS and homogenized for ~1 min using a handheld homogenizer (Kimble/Kontes, New Jersey). This homogenization procedure was repeated for each condition tested. Next, 100 µL of this sample was then spread/plated onto an LB agar plate. To assay subsequent time-points, three flies were placed in 200 µL of PBS and homogenized as above. This sample was then serially diluted, 20 µL into 180 µL of PBS until a dilution of 10^5^ was obtained. Next, 40 µL from each dilution from 100 to 10^5^ was then spot plated onto an LB agar plate and allowed to dry. All plates were incubated at 37 °C overnight before counting. Each in vivo bacterial load experiment was independently repeated three times and each experiment included three replicate samples per time point for all experiments. Error bars were calculated to represent one standard error.

Due to limited bacterial sample availability from the spaceflight mission and the time required to inject 50 or more individual flies per condition per experiment, not all samples (Space 1 and 2, Ground 1 and 2) were included in every experiment. After initial experiments had verified the consistency of the virulence data from Space 1 and Space 2 samples relative to the Ground 1 and Ground 2 samples, only one sample from each spaceflight and ground condition was chosen in subsequent studies.

### Ground subcultures of spaceflight sample

Injections to test the virulence of space-flown bacteria were performed as noted above where the spaceflight and ground bacteria were diluted in PBS from frozen glycerol stocks and directly injected into the fly host. However to test whether the space-flown bacteria would retain their increased virulence after growing under ground conditions for several generations, bacterial cultures were first allowed to grow for 24 h (~23 generations of growth) on the ground before injecting into *Drosophila* hosts. A sterile inoculation loop was used to transfer a small amount of glycerol stocks into liquid LB + 100 µg/mL streptomycin, then grown for 24 h in a 37 °C incubator with a shaker set to 225 rpm. These samples were then diluted in PBS down to ~4 × 10^3^ CFU/mL in sterile PBS for injections, and injections were performed as described above.

### Growth of *S. marcescens* Db11 in the RWV

Cultures of Db11 were grown from non-spaceflight lab stocks of Db11 frozen at −80 °C in Copan Cryovials, onto agar plates with streptomycin (LB + 100 µg/mL) for 18–24 h and then stored at 4 °C for at most 2 weeks. Liquid subcultures were created using a single colony from these agar plates and grown in conical tubes at 37 °C in liquid LB media containing 100 µg/mL streptomycin at 25 rpm for 24 h. These liquid subcultures were then diluted down to A600 of 0.100 in LB media before being further diluted 1:1000 in LB + 100 µg/mL streptomycin and placed into a sterile disposable 10 mL RWV (Synthecon). Air bubbles were removed from the RWV and sealed. Pairs of RWV were then placed in either horizontal (simulated microgravity or LSMMG) or vertical (normal gravity, NG) axis at 37 °C and rotated at 25 rpm (as in Nickerson et al.^11^) Samples were grown for 24 h to ensure that stationary phase was reached.

### Injection of RWV samples into *D. melanogaster*

Injections were performed as noted above with the following alterations. Stationary phase cultures from RWV were diluted down to an A600 of 0.100 in PBS and then further diluted down 1/100 fold to an end concentration of ~8 × 10^3^ CFU/mL in PBS. Approximately 65 nL of sterile PBS, LSMMG, or NG cultures were then injected into 2–3-day old flies as noted above. Survival and bacterial load were both monitored as previously stated.

### RNA extraction and sequencing from infected fly host

In order to see whether there was any difference in host immunity after infection with Space or Ground bacteria, RNA was extracted in triplicate from two live ground-reared adult flies for each treatment (space or ground sample injection) approximately 18 h after infection using the Qiagen RNeasy Mini Kit (catalog no. 74104) with β-mercaptoethanol (10 µL/mL) in the lysis buffer. Lysis was performed by placing the flies in a 1.5 mL tube containing 600 µL of the lysis buffer, which were then homogenized for ~1 min using a handheld homogenizer (Kimble/Kontes, New Jersey). The entire homogenate was then placed in the manufacturer-supplied spin column, where an on-column DNase digestion step was performed as per manufacturer recommendation). An additional purification step was added using additional wash steps with the kit buffers. Samples were sequenced on the Illumina HiSeq 4000 platform with 100 bp reads and paired-end library prep. Transcript abundance estimates from the raw sequenced reads were made using kallisto (version 0.42.4)^68^ and the *D. melanogaster* reference transcriptome (build r6.13). Differential expression analysis between Space and Ground bacteria infection conditions was performed using sleuth (version 0.30.0) Wald test.^69^ Transcripts with *q*-values < 0.05 were considered significantly differentially expressed.

### Statistical analysis

Statistical analysis was performed in GraphPad Prism 7.0 and JMP Pro 13 (SAS). Graphs were made in GraphPad Prism 7.0 and in Excel (version 15.4). For survival analyses, a Cox Proportional Hazards model was used, with the exception of the *Toll* and *Imd* mutant injections, for which a Log-rank Mantel-Cox analysis was performed to compare curves across treatments. The Cox Proportional Hazards model allows for the comparison of survival between all groups when there are multiple risk factors or treatments involved. For in vivo growth measurements, a one-way ANOVA was used in combination with a Tukey-Kramer post hoc analysis. For all statistical analyses with the exception of the first subculture growth and survival, only results with a statistical significance < 0.05 are reported, due to the magnitude of results tables.

### Ethics statement

We use the invertebrate model system *Drosophila melanogaster* for this study, in accordance, the study was exempt from ethics committee approval.

## Supplementary information

Supplementary material

nr-reporting-summary

## Data Availability

The datasets generated during and/or analyzed during the current study are available from the corresponding author on request. The RNA-seq datasets, supporting the conclusions of this article, have been deposited in NCBI's Gene Expression Omnibus and are accessible through GEO Series accession number GSE138116.
