# Supplementary material for: Spaceflight and simulated microgravity conditions increase virulence of Serratia marcescens in the Drosophila melanogaster infection model
Source: NPJ Microgravity. 2020 Feb 4;6:4. doi: 10.1038/s41526-019-0091-2 (PMC7000411; doi:10.1038/s41526-019-0091-2)
Supplement: Supplementary file 1 — Supplementary material [file 41526_2019_91_MOESM1_ESM.pdf]

Supplementary Material

Methods - Bacterial Growth Curves

To initiate the culture, bacteria were taken from the spaceflight or ground control 50% glycerol stocks that were stored at -80^{0}C in 50 µl aliquots, and then placed in a 50 mL subculture containing fresh LB media with 100 µg/mL streptomycin at ~1x10^8 CFU/ml density (as estimated by spectrophotometry). Samples were then grown at 37°C in a shaker rotating at 225 rpm. Estimates of growth (colony forming units) were taken every 2 hours from the above culture for 30 hours total by diluting 20 µl of the sample in 180 µl of fresh LB media, then reading immediately at 600 nm on a NanoDrop 2000c (Thermo Scientific) blanked with the initial measurement. Both Space and Ground treatments were run in triplicate and averaged at each timepoint, and the entire experiment was repeated independently three times with spaceflight and ground samples. Data was analyzed in JMP Pro 12 using an ANOVA with repeated measures.

Results – Bacterial growth curves

In vitro growth was significantly higher in the spaceflight bacteria compared to the ground bacteria of the same strain ($t=4.66$, $P<0.0001$).

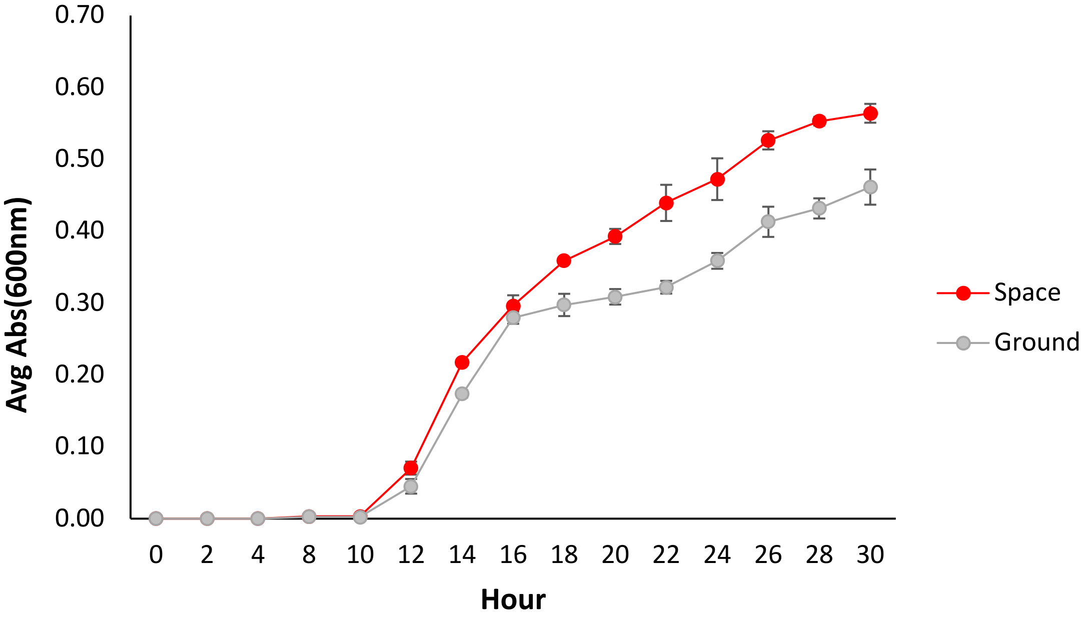

Supplementary Figure 1. Growth curves for spaceflight bacteria compared to ground controls *in vitro*. Spaceflight and ground samples were both stored in 50% glycerol stock and diluted to starting concentrations of ~1x10^8CFU. Growth at 37°C was then measured by taking a sample every 2 hours for 30 hours total, diluting the sample 10x, and measuring ABS at 600nm on a NanoDrop spectrophotometer. Error bars represent 1 standard error.

Supplementary Table 1. Transcript-level differential expression from RNAseq of ground-reared flies infected with either space or ground control *Serratia marcescens*, calculated using sleuth package Wald test. Transcripts with q-values < 0.05 were considered significantly differentially expressed, these are the only values included in this table. All other values were >0.05.

target_id	ens_gene	ext_gene	pval	qval	[-log10(qval)]	beta
FBtr0310667	FBgn0033062	Ars2	8.64E-09	0.00017907	3.746974596	-5.8759894
FBtr0085912	FBgn0000559	eEF2	2.61E-06	0.01355726	1.867828177	-7.2844011
FBtr0088122	FBgn0010357	betaTry	2.62E-06	0.01355726	1.867828177	0.76007008
FBtr0075412	FBgn0014163	fax	2.11E-06	0.01355726	1.867828177	-1.238556
FBtr0305083	FBgn0023172	RhoGEF2	4.92E-06	0.01700121	1.769520087	-7.1835147
FBtr0333107	FBgn0260442	rhea	4.84E-06	0.01700121	1.769520087	5.2835701
FBtr0087437	FBgn0012042	AttA	6.01E-06	0.01779894	1.749605913	4.65161273
FBtr0077042	FBgn0035666	Jon65Aii	7.16E-06	0.01853873	1.731919905	0.87658333
FBtr0089350	FBgn0283521	lola	8.76E-06	0.01859388	1.730629941	7.20501561
FBtr0076398	FBgn0045823	vsg	8.98E-06	0.01859388	1.730629941	6.99749765
FBtr0079035	FBgn0031673	CG31650	2.58E-05	0.04856692	1.31365946	-6.1667293

Supplementary Table 2. Statistical output for proportional hazards analysis of dif^{1} mutant *D. melanogaster* line after infection with ground and space bacteria samples.

Level 1	Level 2	Risk Ratio	P>ChiSq
Space	Ground	28.63	<0.0001
Space	12.5% Glycerol	75.5	<0.0001
Ground	12.5% Glycerol	1.89	0.052

Supplementary Table 3. Statistical output for proportional hazards analysis of PGRP-SA^{seml} mutant *D. melanogaster* line after infection with ground and space bacteria samples.

Level 1	Level 2	Risk Ratio	P>ChiSq
Space	Ground	14.6	<0.0001
Space	12.5% Glycerol	45.51	<0.0001
Ground	12.5% Glycerol	2.11	0.068

Supplementary Table 4. Statistical output for proportional hazards analysis of PGRP-LC$^{\Delta 5}$ mutant *D. melanogaster* line after infection with ground and space bacteria samples.

Level 1	Level 2	Risk Ratio	P>ChiSq
Space	Ground	8.62	<0.0001
Space	12.5% Glycerol	13.6	<0.0001
Ground	12.5% Glycerol	8.22	<0.0001

Supplementary Table 5. Statistical output for proportional hazards analysis of *Imd*^{1} mutant *D. melanogaster* line after infection with ground and space bacteria samples.

Level 1	Level 2	Risk Ratio	P>ChiSq
Space	Ground	3.04	0.036
Space	12.5% Glycerol	14.6	<0.0001
Ground	12.5% Glycerol	11.21	<0.0001

Supplementary Table 6. Statistical output for proportional hazards analysis of *rel*^{E20} mutant *D. melanogaster* line after infection with ground and space bacteria samples.

Level 1	Level 2	Risk Ratio	P>ChiSq
Space	Ground	3.67	0.027
Space	12.5% Glycerol	16.44	<0.0001
Ground	12.5% Glycerol	9.7	<0.0001
